# Perceptions of Weight and Health Practices in Hispanic Children: A Mixed-Methods Study

**DOI:** 10.1155/2015/761515

**Published:** 2015-08-25

**Authors:** Byron Alexander Foster, Daniel Hale

**Affiliations:** Department of Pediatrics, University of Texas Health Science Center at San Antonio, 7703 Floyd Curl Drive MC 7803, San Antonio, TX 78229, USA

## Abstract

*Background.* Perception of weight by parents of obese children may be associated with willingness to engage in behavior change. The relationship between parents' perception of their child's weight and their health beliefs and practices is poorly understood, especially among the Hispanic population which experiences disparities in childhood obesity. This study sought to explore the relationship between perceptions of weight and health beliefs and practices in a Hispanic population. *Methods.* A cross-sectional, mixed-methods approach was used with semistructured interviews conducted with parent-child (2–5 years old) dyads in a primarily Hispanic, low-income population. Parents were queried on their perceptions of their child's health, health practices, activities, behaviors, and beliefs. A grounded theory approach was used to analyze participants' discussion of health practices and behaviors. *Results.* Forty parent-child dyads completed the interview. Most (58%) of the parents of overweight and obese children misclassified their child's weight status. The qualitative analysis showed that accurate perception of weight was associated with internal motivation and more concrete ideas of what healthy meant for their child. *Conclusions.* The qualitative data suggest there may be populations at different stages of readiness for change among parents of overweight and obese children, incorporating this understanding should be considered for interventions.

## 1. Introduction

Obesity continues to be a major public health issue with ethnic and racial minorities disproportionately affected [1]. Parental engagement has been recognized as necessary for any successful intervention around weight in early childhood. Studies examining perceptions of weight have found that parents of overweight and obese children consistently misclassify their child's weight as normal [2–6], potentially making behavior change even more challenging without recognition of the problem.

Parents of younger children tend to underestimate the degree of overweight and have less concern about their child's weight relative to parents of older children [3]. In one study, only 1.9% and 17.1% of overweight and obese children, respectively, perceived their child as overweight [7]. Similarly, mothers of young children incorrectly perceived their overweight or obese children to be “about the right weight” 93.6% and 77.5% of the time, respectively [8]. A recent systematic review and meta-analysis that included children of all ethnic backgrounds found that significant predictors of weight underestimation among overweight and obese children were younger age and lower body mass index (BMI) [9].

Importantly, these weight perceptions may have an impact on behaviors and weight outcomes; children of mothers who underestimate their child's weight have been found to have greater weight gain in early childhood [10]. Another study showed that despite underestimating weight relative to the standard pictogram, weight perception did correlate with BMI [11]. Parents' accurate perception of their child's weight has been associated with a greater readiness for change [12]. Finally, historical data show that there is a greater extent of underestimation of weight in overweight and obese children now compared with 25 years ago [13].

There are limited data around why parents of overweight and obese children consistently underestimate their children's weight status. Studies using qualitative methods have identified strong cultural preferences for heavier children, particularly among Mexican-American mothers [14]. Acculturation within Mexican-American parents may also play a role with those less acculturated to the United States perceiving heavier children to be healthier [15]. Finally, a different focus on health with feeding being highly valued and weight status, particularly in the young, being of a lower priority has been identified [16].

In south Texas, with a majority Latino population and early childhood obesity being at levels higher than the national average [17], understanding how perceptions of weight relate to health behaviors and practices has implications for the design and implementation of obesity interventions. The associations between perceptions of weight and healthy behaviors and parental attitudes towards weight have not been examined. To address this gap, we explored the attitudes and reported behaviors of parents of overweight and obese children, and we used a qualitative approach to examine whether perceptions of weight status had any influence on parental attitudes and behaviors related to their child's health.

## 2. Methods

### 2.1. Design and Sample Population

Subjects were recruited from the waiting rooms of two pediatric clinics in Harlingen, Texas, which is located in Cameron County on the border with Mexico in the lower Rio Grande valley. Parent-child dyad pairs were recruited if their child was between 24 and 72 months of age and could speak English. We included children from any weight category in order to compare perceptions of weight across the weight spectrum. Exclusion criteria were a diagnosis of moderate to severe developmental delay, seizure disorder, diabetes, or any genetic diagnosis or cerebral palsy. The children were weighed without shoes but in their normal clothes using a portable scale, and height was measured using a stadiometer. After enrollment, parent-child dyads were interviewed using a semistructured questionnaire in their homes by two trained staff of the research team. Interviews were conducted between March and October 2013.

### 2.2. Ethics

This study was approved by the institutional review board of the University of Texas Health Science Center at San Antonio and all parents consented to participate. Parents were given a gift card to a local grocer on completion of the study.

### 2.3. Questionnaire

The questionnaire was designed with open-ended questions around perceptions of health and weight (Supplemental File 1 in Supplementary Material available online at http://dx.doi.org/10.1155/2015/761515). Parents were asked to describe their day with their child and queried on the reasons, if any, behind why they did what they did with their child. Specific standardized questions were used for ethnicity, income, education, family size, parental characteristics (age, height, and weight), dietary intake using a single-pass 24-hour recall, and activity and screen time habits of the child reported by the parent, as previously published [18]. Participants were asked specifically if they thought their child was at a healthy weight. Further, perception of weight was assessed using a standardized pictogram depicting children of the appropriate sex with a median weight child displayed along a continuum with heavier and lighter children with a total of 7 child sizes (Supplemental File 2). These pictograms have been used previously to assess parental perceptions of weight [3]. Parents were asked to circle the pictogram which looked most like their child's current size, and then, on a separate pictogram scale, what they would like their child's body to look like, if at all different. The questionnaire was administered by two trained research staff with an audio recording and then professionally transcribed.

### 2.4. Analysis

#### 2.4.1. Grouping Participants

Participants were classified as overweight if their body mass index (BMI) percentile was between the 85th and 95th percentiles and obese if their BMI percentile exceeded the 95th percentile for age and sex (Table 1). We also categorized the subjects based on the parental responses to the two pictogram questions about perceived current weight and perceived ideal weight while taking into account their actual measured weight status (Table 2). For the overweight and obese children, we subdivided them by whether their parent had indicated that their ideal weight was less than their current weight or if the parent wanted them to either stay the same or be heavier. For example, obese children, defined by their BMI percentile, were separated into two groups: obese children whose parents indicated an ideal weight larger than or the same as their current weight, and obese children whose parents indicated an ideal weight smaller than their current weight. We then examined whether these two groups showed any differences in health behaviors or attitudes.

#### 2.4.2. Qualitative Analysis

The transcripts were read first by the primary interview team to assess for quality and errors. The research team then read each interview line by line at least twice to elicit themes and develop a coding scheme. We used a grounded theory approach to the analysis, with open coding used. First, each interview was read by two research team members independently and sections were coded using mixed emic and etic open coding. After all the interviews were coded, each individual interview was coded combining each set of codes from each team member via discussion, at which point themes and subthemes across the interviews began to emerge. Then, another analysis was done both within weight based groups and across all groups, and the themes and subthemes were resorted and recoded. The same two staff conducted all of the interviews, reducing variability in data collection.

#### 2.4.3. Quantitative Analysis

Quantitative data including demographic variables were extracted from the interviews with descriptive analyses done appropriately with chi-square or *t*-tests using SPSS 21.0 (IBM, USA). The demographics of this sample have been previously described [18].

## 3. Results

### 3.1. Sample Characteristics

Our population included 40 parent-child pairs; the subset of 24 overweight and obese children, the focus of this study, did not differ in demographics from the normal weight subjects (Table 1). In the overweight and obese group, 9 subjects were overweight and 15 were obese. The majority were of Hispanic ethnicity and had a relatively low income, and the mean BMI of parents was in the obese category (Table 1).

### 3.2. Weight Perception and Health Practices

Of the overweight and obese children, 12 of 24 of their parents perceived their ideal weight as less than their current, while the other half perceived their ideal weight as the same or larger than the current one (Table 2). The BMI percentile of children whose parents perceived their ideal weight as less was higher than their peers (*p* = 0.01) and they consumed less juice (*p* = 0.02).

### 3.3. Qualitative Results

The qualitative analysis examining health perceptions revealed three main themes: reasons my child is or is not healthy, why some foods are good or bad for children, and how to make a child healthy.

#### 3.3.1. My Child Is or Is Not Healthy Because

Most parents of overweight and obese children had similar descriptions of what a healthy child should be, with being active emerging as a strong theme (18 of 24 parents of overweight or obese children reported some version of being active) in support of a positive health perception.

*To me he's fine. I do not see him unhealthy. I do not – he's very – he's a little boy who is very happy and energetic. He's running around. To me he's not obese…he's a big boy for his age. OB8*

When discussing whether their own child was healthy, parents often stated at least one positive thing their child does (eats some fruits, runs around a lot) before discussing whether they thought their child was healthy. In discussing weight, many parents of the overweight and obese children brought up the concept of “not fat” or “not obese” despite no phrasing in the questions using those terms (10 of 24 parents). A parent indicated no difference between ideal and current weight:

*Because I mean, I do not really think he's fat. He's just a bigger kid. Like he's more – he's just thick, you know. He's real heavy. He's always been that way, just like the baby. OW3*

A parent indicated a lower ideal weight than her child's current weight:

*I do not think that he's overweight. I think he's okay, but if I keep doing what I'm doing, he's eventually going to get unhealthy. If I keep doing – letting him get his way and giving him snacks before meals, it's not going to be good for him. OW11 *

Parents across the spectrum of perceived current versus ideal weight expressed a significant reliance on what they thought the doctor said or did not say regarding their child's weight (14 of 24 parents). However, only parents of children who indicated their child's ideal weight to be smaller than their current perceived weight described the doctor indicating a problem:

*The doctors are like she's a little bit overweight. She's not at the weight I would like. It used to be they would say, oh, she's weighs this much, do you want to see a dietician? Just watch what she eats, make sure she stays active and stuff like that. OB18*

Other parents from the same doctor's office expressed that since the doctor had not said anything, their child was healthy. This parent indicated no difference between current and ideal weight:

*The doctor has not told me otherwise yet. There are days that I do think that maybe she is a little bit overweight, but the doctor has not told me anything. OB14 *

#### 3.3.2. Good versus Bad Foods

In describing why some foods are better than others, the group that indicated a desire for weight reduction was much more specific in their rationales (8 of 12 parents spontaneously provided a specific rationale):

*Vegetables have a lot of benefits as far as helping you with your eyes, and your teeth, and your bones, and stuff like that. OB9*

*Water because it does not contain no sugars. As long as it's purified water or whatever, it should be safe for anybody. And milk because it helps with their bones…. OB12*

A mother indicated some frustration in implementing her knowledge:

*Good foods are…vegetables, fruits, anything that's boiled…because they do not have oil and grease and much calories. Like boiled rice and stuff like that, and my girls do not like that. OB15*

Those parents who did not perceive a difference between their child's current weight and ideal weight had more vague descriptions; 6 of 8 parents who indicated their child should be larger make references to some external authority:

*Good foods are fruit, vegetables, chicken…because that's what I hear. OB20*

*Vegetables and meat protein are good - because that is what we're told is good for them. OB3*

#### 3.3.3. How to Make a Child Healthier

Parents who indicated a lower ideal weight than their child's current weight described more specific strategies that they would like to put into place:

*Just limit everything, and if they are not wanting to eat the healthy foods I try to mix it up like apple slices with caramel like the little dips or just give them so they can eat it. Like carrots there are some that do not carrots but you put like the ranch dip or the peanut butter, they go ahead and eat it because it's a mix and you're still eating. OW5*

Frustration with the strategies not working or not being implemented was expressed as well:

*If I could, feed them better and more and better exercise and maybe more planned – a planned routine. You know, I know by – sit down and plan a schedule like he needs to eat this and he needs to do this and that. I'm sure if I had the means to do that. But will it happen is a whole other thing. You know what I mean? I'm not that disciplined, you know what I mean, myself. OB5*

Some parents indicated no difference between ideal and current weight:

*I think anything is good for them like as long as they eat and they're healthy, I think anything is good. I do not have, no, you cannot eat this. All foods are good for him…Just not too much of it. OW7 Do not feed them many sugary items, and do not eat a lot of greasy, take-out food. Eat more vegetables, more fruit. OB20*

Essentially two groups from among the overweight and obese groups emerged from the qualitative analysis: one group that verbalized strategies and habits they would like to implement to make their child healthier but that they had tried and failed at and a second group that expressed less specific strategies to become healthier and expressed ideas of health in more abstract terms. For the most part, these two groups tended to align with the two weight perception groups; that is, those who perceived a difference between ideal and current body weight for their child also expressed more specific strategies.

### 3.4. Weight Perception of Parents

Parents of overweight and obese children misclassified their child's current weight status as normal or underweight 58% of the time using the pictogram scale. Similarly, 67% of parents of overweight or obese children responded in the affirmative when asked directly if their child was at a healthy weight. We also examined the overweight or obese children by whether their parents indicated a difference between their perceived current weight and ideal weight based on pictograms. Of parents who marked their child's ideal weight as lighter than their current, 72% perceived their child's current weight as larger than the median depiction. Of parents who saw no difference in perceived current weight versus ideal weight, 50% indicated their child was at more than the median weight on the pictogram scale, and only 25% of parents who wanted their child heavier indicated their child's current weight was larger than the median. In comparison, 38% of normal weight children's parents wanted them to ideally be heavier, and none chose a picture of a child greater than the median weight.

## 4. Discussion

The qualitative data on attitudes and beliefs around health practices differed by the parent's perception of their child's weight. Among overweight and obese children from a largely Hispanic sample in south Texas, parents had a wide range of perceptions of their child's weight. Overall, a more accurate perception of their child's current weight was associated with indicating a smaller ideal weight using a pictograph scale. Parents who indicated a difference in ideal weight versus current weight described more specific knowledge related to health and health practices as well as more specific preparation or planning for implementing healthier habits; however, they did not differ on most health practices related to diet or activity. These two different populations within the same overall excess weight category would be expected to respond differently to healthy habits or weight-targeted interventions.

Previous studies have documented that perception of weight in early childhood is strongly influenced by functional limitations [3, 19, 20]; in other words, if the child does not appear markedly larger than their peers and is not out of breath, they are fine. This is consistent with our finding that, among parents of overweight and obese children, a healthy child is described as an active, happy child. The primary discussion linking weight and health at this age was in discussing how their weight was not a health problem, using terms such as “big” or “heavy.”

The misclassification of weight status in this study was similar to estimates of others [6], particularly when taking into account our participants' relatively low education and income which have been correlated with misclassification of weight previously [4]. The finding of parents misclassifying the weight status of their overweight and obese children is not new [21]. However, the interesting aspect of this study was the relationship between parents' perceptions of their child's current weight status and their desire for their child to lose weight.

Studies are mixed on the influence of physicians' advice with some reporting that the provider's input was viewed as important helping to identify weight issues before they are visually obvious [22] while others have described a rejection of the doctor's assessment [19]. Our study documented the importance of the doctor's input for a subset of patients, all of whom came from the same practices making it less likely that the variability lies in the doctor, though what is heard during a doctor's visit has complex interactions.

A longitudinal cohort study found that accurate weight perception at age 5 was a positive predictor of higher future BMI implying that accurate perception alone is not enough to make positive changes [23]. The qualitative differences in described reasons for certain practices with some parents describing more concrete and internalized rationales are similar in concept to motivational interviewing which has been explored recently in pediatric obesity research [24–26]. Motivation is an important aspect of any behavioral change. Further investigation into whether interventions to address perceptions of weight among overweight and obese Hispanic children may influence their subsequent motivation and behaviors is warranted as our sample was cross-sectional.

This work has limitations of sample size and external validity as we examined a specific population in south Texas, and we were only able to interview English-speaking subjects. However, the rich qualitative data adds significant value to health perceptions in a population with some of the highest rates of obesity in the United States.

## 5. Conclusions

This study adds to prior work by describing the relationship between perceived weight status and ideal weight status and outlines how parents of overweight and obese children have different views of their child's health related to their perceived weight status. These findings have potential application to both clinical research and clinical practice should they be replicated in a larger sample. In clinical practice, understanding the parent's perception of their weight in some objective or structured way would help guide the counseling and education of that parent as parents who do not see their child's current weight as abnormal may not be receptive to strategies of addressing it. Furthermore, in clinical research, an assessment of weight perception prior to enrolling subjects in an obesity research intervention could assist in predicting compliance and outcomes and potentially provide stratification strategies for educational or behavioral interventions.

## Supplementary Material

Supplemental File 1 is the basic structure and outline of questions used in the semi-structured interview done with participants that explored their perceptions of weight and health. Supplemental File 2 is the figure used to depict the various weights of children as a supplemental method of assessing parental perception of weight.

## Figures and Tables

**Table 1:** Demographic characteristics of the subjects by weight category.

	Normal weight (*n* = 16)	Overweight and obese (*n* = 24)	*p* value
Child's age in months, mean (SD)	45.6 (12.5)	46.6 (11.5)	0.79
Sex, % male (*n*)	62.5 (10)	45.8 (11)	0.35
Ethnicity, % Hispanic (*n*)	93.8 (15)	87.5 (21)	0.64
Language at home, % English (*n*)	75.0 (12)	83.3 (20)	0.72
Education			0.51
% high school or less (*n*)	31.3 (5)	45.8 (11)	
% any college or higher (*n*)	68.8 (11)	54.2 (13)	
Income, % (*n*)			0.12
<100% FPL	43.8 (7)	69.6 (16)	
100–200% FPL	31.3 (5)	26.1 (6)	
>200% FPL	25.0 (4)	4.3 (1)	
BMI of parent, mean (SD)	30.1 (5.2)	32.8 (8.4)	0.25
Child's BMI percentile, mean (SD)	56.1 (19.5)	95.7 (4.4)	<0.001
Family size, mean (SD)	5.2 (1.4)	4.8 (1.4)	0.43

SD = standard deviation; FPL = federal poverty line; BMI = body mass index.

**Table 2:** Health practices among overweight and obese subjects grouped by their parents' comparison of their ideal weight compared to their current weight.

	Should be smaller (*n* = 12)	No change or should be larger (*n* = 12)	*p* value
BMI of parent, mean (SD)	31.8 (6.2)	33.8 (10.3)	0.56
BMI percentile of child, mean (SD)	97.9 (2.6)	93.6 (4.9)	0.01
Fruit and vegetable servings, mean (SD)	2.8 (1.6)	3.0 (1.5)	0.70
Water per day in ounces, mean (SD)	20 (11)	39 (43)	0.23
Soda per day in ounces, mean (SD)	11 (9)	5 (6)	0.15
Juice per day in ounces, mean (SD)	11 (6)	20 (9)	0.02
Milk per day in ounces, mean (SD)	16 (10)	16 (11)	0.95
Screen time per day in hours, mean (SD)	3.3 (1.7)	2.4 (2.2)	0.28
Activity per day in hours, mean (SD)	1.8 (1.3)	1.6 (1.3)	0.79

SD = standard deviation; BMI = body mass index.
