# Supplementary figures and images for: Perceptions of Weight and Health Practices in Hispanic Children: A Mixed-Methods Study
Source: Int J Pediatr. 2015 Aug 25;2015:761515. doi: 10.1155/2015/761515 (PMC4561986; doi:10.1155/2015/761515)

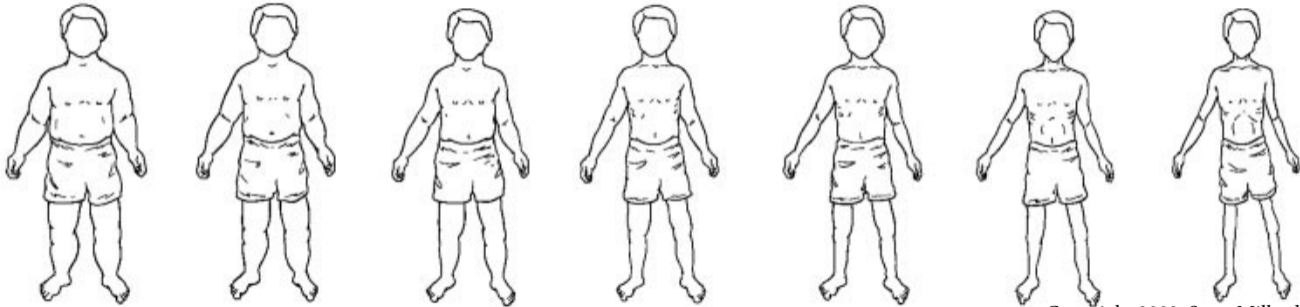

Copyright 2003, Scott Millard

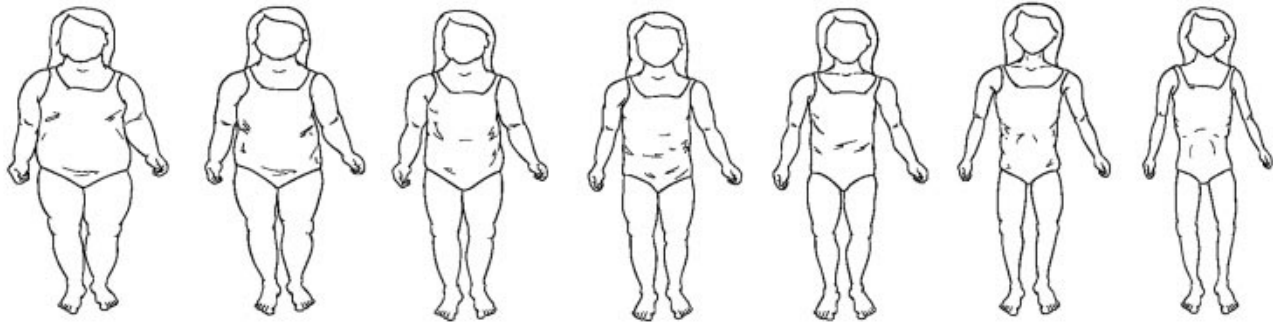

Copyright 2003, Scott Millard

Supplement: Supplementary file 1 — Supplemental File 1 is the basic structure and outline of questions used in the semi-structured interview done with participants that explored their perceptions of weight and health. Supplemental File 2 is the figure used to depict the various weights of children as a supplemental method of assessing parental perception of weight. [file 761515.f1.pdf]
